# First-in-Human Safety, Imaging, and Dosimetry of a Carbonic Anhydrase IX–Targeting Peptide, [^68^Ga]Ga-DPI-4452, in Patients with Clear Cell Renal Cell Carcinoma

**DOI:** 10.2967/jnumed.123.267175

**Published:** 2024-05

**Authors:** Michael S. Hofman, Ben Tran, Darren R. Feldman, Anna Pokorska-Bocci, Solen Pichereau, Jonathan Wessen, Mohammad B. Haskali, Richard B. Sparks, Olena Vlasyuk, Ivana Galetic

**Affiliations:** 1 Peter MacCallum Cancer Centre, Melbourne, Victoria, Australia;; 2 Sir Peter MacCallum Department of Oncology, University of Melbourne, Melbourne, Victoria, Australia;; 3 Memorial Sloan Kettering Cancer Center, Weill Cornell Medical College, New York, New York;; 4 Debiopharm International SA, Lausanne, Switzerland; and; 5 CDE Dosimetry Services, Inc., Knoxville, Tennessee

**Keywords:** theranostic, PET/CT, clear cell renal cell carcinoma, [^68^Ga]Ga-DPI-4452

## Abstract

[^68^Ga]Ga-DPI-4452, a first-in-class carbonic anhydrase IX–binding radiolabeled peptide, is the imaging agent of a theranostic pair with [^177^Lu]Lu-DPI-4452, developed for selecting and treating patients with carbonic anhydrase IX–expressing tumors. Here, [^68^Ga]Ga-DPI-4452 imaging characteristics, dosimetry, pharmacokinetics, and safety were assessed in 3 patients with clear cell renal cell carcinoma. **Methods:** After [^68^Ga]Ga-DPI-4452 administration, patients underwent serial full-body PET/CT imaging. Blood and urine were sampled. Safety was monitored for 7 d after injection. **Results:** Tumor uptake was observed at all time points (15 min to 4 h). Across 36 lesions, the SUV_max_ at 1 h after administration ranged from 6.8 to 211.6 (mean, 64.6 [SD, 54.8]). The kidneys, liver, and bone marrow demonstrated low activity. [^68^Ga]Ga-DPI-4452 was rapidly eliminated from blood and urine. No clinically significant toxicity was observed. **Conclusion:** [^68^Ga]Ga-DPI-4452 showed exceptional tumor uptake in patients with clear cell renal cell carcinoma, with very high tumor-to-background ratios and no significant adverse events, suggesting potential diagnostic and patient selection applications.

## Visual Abstract

**Figure:**
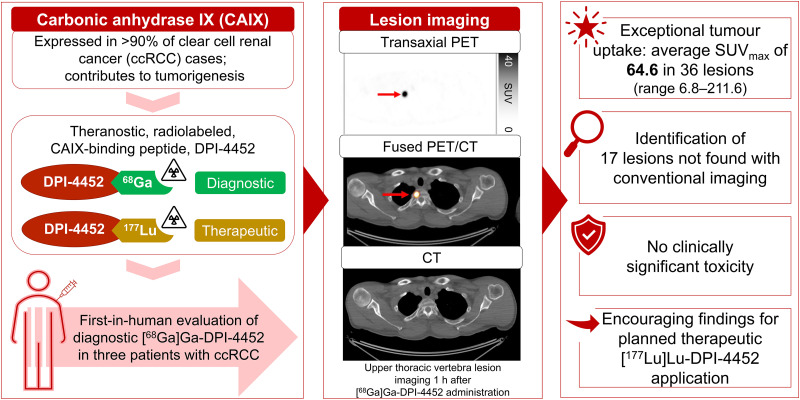

Clear cell renal cell carcinoma (ccRCC) accounts for 70%–80% of renal cell carcinoma cases (1). Localized disease can be cured with surgery and ablative therapy regimens; however, one third of patients present with or develop metastases, with poor prognosis (2). Despite major advances in treatment for renal cell carcinoma, the 5-y relative survival rate for distant metastatic disease remains low (3,4).

Carbonic anhydrase IX (CAIX) is a cell-surface glycoprotein involved in acid–base regulation. Aberrant tumor expression contributes to extracellular acidification, promoting tumor progression (5,6). The CAIX-encoding gene is overexpressed in more than 90% of ccRCC cases, often because of alterations in the von Hippel–Lindau gene (7,8). With physiologic CAIX expression restricted to gastrointestinal epithelia (9), high tumoral expression presents diagnostic and therapeutic opportunities. Indeed, PET/CT-based tumor visualization with ^89^Zr-labeled anti-CAIX antibodies ([^89^Zr]Zr-girentuximab) can aid diagnosis of localized and metastatic ccRCC and enable differentiation of indolent versus benign tumors, which is challenging with conventional imaging (10–12).

The CAIX-binding cyclic peptide DPI-4452, labeled with diagnostic (^68^Ga) or therapeutic (^177^Lu) radioisotopes, provides a novel theranostic pair to target CAIX-expressing tumors. Here, we report the characteristics of diagnostic [^68^Ga]Ga-DPI-4452 in patients with ccRCC.

## MATERIALS AND METHODS

### Patient Population and Study Design

This first-in-human, open-label, nonrandomized, multicenter phase 1/2 study (NCT05706129) aims to assess the safety, tolerability, and imaging characteristics of [^68^Ga]Ga-DPI-4452 (part A) and the safety, efficacy, and therapeutic potential of [^177^Lu]Lu-DPI-4452 (parts B and C) in patients with unresectable locally advanced or metastatic solid tumors (Supplemental Fig. 1; supplemental materials are available at http://jnm.snmjournals.org) (13,14).

Results from the completed ccRCC imaging cohort (part A) are reported. Patients with histologically confirmed unresectable, locally advanced or metastatic ccRCC must have received at least 2 lines of treatment in the metastatic setting (including a tyrosine kinase inhibitor and an immune checkpoint inhibitor). Further inclusion and exclusion criteria are in the supplemental methods.

The primary objective of part A was to evaluate the safety and tolerability of a single intravenous injection of [^68^Ga]Ga-DPI-4452. Secondary objectives included establishment of optimal imaging procedures for positive lesions, biodistribution assessment, dosimetry, pharmacokinetics, and concordance between [^68^Ga]Ga-DPI-4452 PET imaging and conventional imaging. The study was institutional review board–approved, and patients provided written informed consent.

### Administration of [^68^Ga]Ga-DPI-4452

DPI-4452 contains a 1,4,7,10-tetraazacyclododecane-1,4,7,10-tetraacetic acid cage allowing chelation with radionuclides. [^68^Ga]Ga-DPI-4452 solutions were manufactured at the site radiopharmacy unit per local regulations before quality control testing and administration. Patients received 185 MBq (±20%) of [^68^Ga]Ga-DPI-4452 as a slow intravenous injection.

### Imaging and Dosimetry

Patients underwent whole-body PET/CT at 15 min, 1 h, 2 h, and 4 h after [^68^Ga]Ga-DPI-4452 administration. Determination of tumor lesions was based on SUV_max_ and SUV_mean_. Regions of interest were drawn on PET/CT images over critical organs and tumor lesions to generate time–activity curves, calculate tumor-to-background ratios per time point, and assess radioactivity residence times.

Dosimetry assessments were based on time–activity curves and time-integrated activity coefficients to determine effective dose and absorbed dose per organ (supplemental methods).

### Pharmacokinetics

Blood and urine were collected before, and at time points up to 6 h after, [^68^Ga]Ga-DPI-4452 dosing and were analyzed onsite with a calibrated γ-counter.

### Safety Assessment

Safety and tolerability were assessed up to 7 d after [^68^Ga]Ga-DPI-4452 administration (supplemental methods).

## RESULTS

Three patients with metastatic ccRCC, aged 48, 51, and 54 y, were enrolled in a single center. All patients had an Eastern Cooperative Oncology Group performance status of 1 or less and had received at least 2 prior lines of systemic therapy (Supplemental Table 1). The mean administered [^68^Ga]Ga-DPI-4452 activities were 174, 198, and 198 MBq.

**FIGURE 1.**
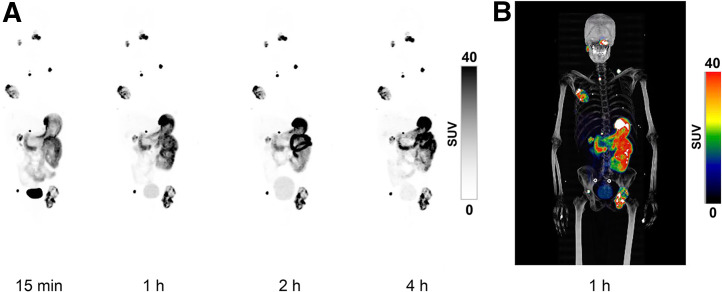
Whole-body maximum-intensity projections over time after [^68^Ga]Ga-DPI-4452 administration. (A) Representative PET images at 4 postadministration time points. (B) PET/CT image at 1 h to allow visualization of anatomic contour.

**FIGURE 2.**
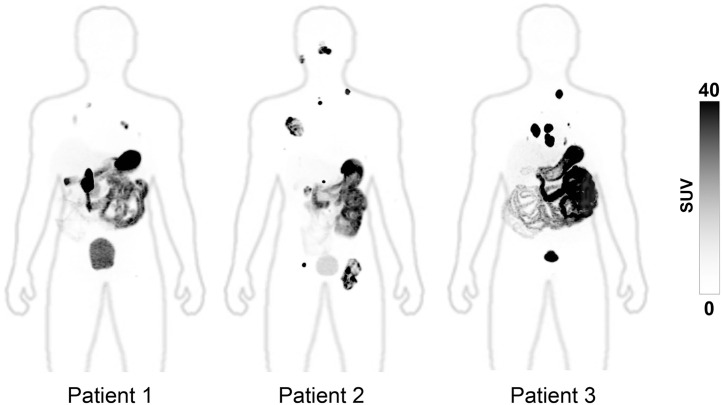
Whole-body maximum-intensity PET projections from 3 patients with ccRCC 1 h after administration of [^68^Ga]Ga-DPI-4452. High tumor-to-background contrast is seen, with background physiologic uptake in stomach, small bowel, and bladder. Partially transparent maximum-intensity projections of corresponding CT scans are overlaid on PET images to allow visualization of anatomic contour.

**FIGURE 3.**
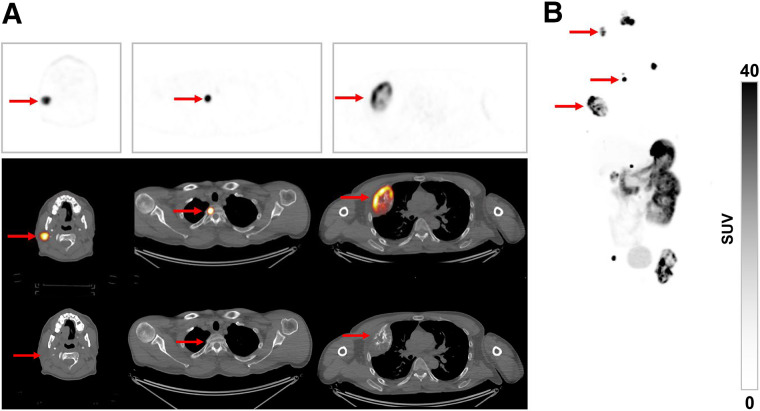
Representative images from patient with ccRCC 1-h after [^68^Ga]Ga-DPI-445 administration. (A) Transaxial PET images (top), fused PET/CT images (middle), and CT images (bottom) showing tracer uptake in tumors (arrows) in parotid gland, upper thoracic vertebra, and chest wall (left to right). (B) Anterior maximum-intensity projection of whole-body image.

After [^68^Ga]Ga-DPI-4452 administration, high tumor-specific uptake was observed as early as 15 min and was sustained for all time points assessed (Fig. 1). One hour was chosen as the optimal time point on the basis of central reader visual assessment of image quality, visualization of all lesions, and heterogeneity in tumor uptake (Fig. 2). Among all lesions, 17 metastases in bone, lymph nodes, lungs, pancreas, and parotid glands were not readily identifiable by conventional imaging (Fig. 3; Table 1). Low renal parenchymal uptake enabled identification of renal tumors. SUV_max_ 1 h after administration across 36 lesions ranged from 6.8 to 211.6 (mean, 64.6 [SD, 54.8]) (Table 2). In patients 1, 2, and 3, the highest SUV_max_ was 109, 106, and 212, respectively, whereas the highest SUV_mean_ was 39, 62, and 89, respectively.

**TABLE 1.** Comparison of PET with PET/CT 1 Hour After [^68^Ga]Ga-DPI-4452 Administration

Finding	Patient 1	Patient 2	Patient 3
Lesions detected by CT and PET	5	6	8
Discordant lesions (not detected by PET)	1*	0	0
Lesions found by PET only	0	8	9
Lesion SUV_max_ range	9–109	7–106	9–212

* Lesion found in lung.

**TABLE 2.** Lesion Uptake of [^68^Ga]Ga-DPI-4452 by Tumor Location 1 Hour After Administration

	Lesions detected by PET only		Nodal lesions found by CT and PET		Nonnodal lesions found by CT and PET		Total lesions detected by CT and PET	
Location	*n*	Mean SUV_max_	*n*	Mean SUV_max_	*n*	Mean SUV_max_	*n*	Mean SUV_max_
Overall	17	42.24 (7.67–106.06)	2	211.61 (211.61–211.61)	17	69.76 (6.83–158.63)	36	64.65 (6.83–211.61)
Adrenal gland					1	109.49 (109.49–109.49)	1	109.49 (NA)
Bone	6	61.26 (16.39–106.06)			3	56.61 (25.31–82.83)	9	59.71 (16.39–106.06)
Chest wall					1	72.96 (72.96–72.96)	1	72.96 (NA)
Kidney					6	104.48 (39.35–158.63)	6	104.48 (39.35–158.63)
Lung*	4	13.29 (7.67–20.11)			5	19.64 (6.83–45.35)	9	16.82 (6.83–45.35)
Lymph node	4	27.9 (13.62–45.93)	2	211.61 (211.61–211.61)			6	89.14 (13.62–211.61)
Pancreas	2	72.83 (46.26–99.40)					2	72.83 (46.26–99.40)
Parotid gland	1	40.15 (NA)					1	40.15 (NA)
Thyroid gland					1	108.53 (NA)	1	108.53 (NA)

* One additional lesion was found with CT imaging only.

NA = not applicable.

Data in parentheses are ranges.

**FIGURE 4.**
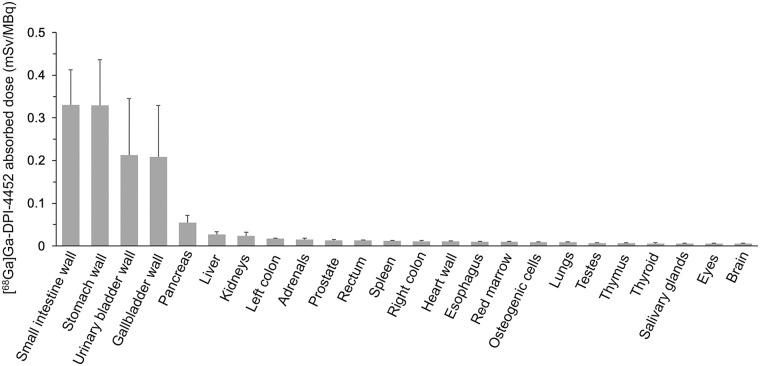
Organ-absorbed doses of [^68^Ga]Ga-DPI-4452 in 3 patients with ccRCC. Error bars represent SD.

OLINDA dosimetry estimates were calculated for 24 organs. Those with the highest mean absorbed doses included the small intestine wall (0.33 [SD, 0.08] mGy/MBq), stomach wall (0.33 [SD, 0.10] mGy/MBq), and gallbladder wall (0.21 [SD, 0.12] mGy/MBq), with a mean whole-body effective dose of 0.06 [SD, 0.02] mSv/MBq. Absorbed doses in the kidney, liver, and bone marrow were low (Fig. 4; Supplemental Table 2).

[^68^Ga]Ga-DPI-4452 blood activity concentration rapidly decreased over time; more than 80% of the total administered radioactivity cleared from the bloodstream within 1 h (Supplemental Fig. 2). The average percentage of the injected dose found in urine ranged from 6.1 (SD, 3.6) to 13.3 (SD, 4.5) across the intervals of 0–1.5 h and 1.5–6 h (Supplemental Fig. 3).

No clinically significant toxicity was observed; treatment-emergent adverse events (headache and increased blood creatine kinase [1 each; 33.3%]) were not causally related to [^68^Ga]Ga-DPI-4452. No significant changes in vital signs, laboratory assessments, or electrocardiograms were observed.

## DISCUSSION

[^68^Ga]Ga-DPI-4452 administration in patients with ccRCC offered outstanding imaging from the earliest (15 min) time point, with sustained tumor uptake up to 4 h and rapid systemic elimination. This is consistent with a nonclinical evaluation of radiolabeled DPI-4452 in which sustained tumor retention (≤48 h after administration) of ^111^In-labeled-DPI-4452 in mice bearing CAIX-positive tumors was observed (15).

PET revealed an exceptionally high tumor-to-background ratio, with background tissues nearly invisible. One-hour after administration was the optimal time point for lesion assessment; this is substantially shorter than the 3–7 d needed with [^89^Zr]Zr-girentuximab in the phase 3 ZIRCON study (11). [^68^Ga]Ga-DPI-4452 also identified 17 metastatic lesions not found with conventional imaging.

No significant adverse events or safety signals arose from [^68^Ga]Ga-DPI-4452 administration. Some radiolabeled peptides have high uptake in the liver or kidneys (16); however, organs receiving the highest absorbed [^68^Ga]Ga-DPI-4452 doses were the small intestine, stomach, and gallbladder walls (consistent with CAIX expression (9)) and the urinary bladder (because of renal elimination). Although [^68^Ga]Ga-DPI-4452 demonstrated a degree of distribution similar to [^89^Zr]Zr-girentuximab (e.g., in the small intestine, stomach, and gallbladder) (17), [^68^Ga]Ga-DPI-4452 had lower uptake in critical organs, including the kidneys, liver, heart wall, and bone marrow. Myelotoxicity is common with multiple cycles of [^177^Lu]Lu-labeled CAIX-targeting antibody treatment (18); however, here, consistent with similar radiolabeled peptide-based imaging agents (19), [^68^Ga]Ga-DPI-4452 showed low bone marrow absorption, possibly indicating low myelotoxicity potential.

## CONCLUSION

[^68^Ga]Ga-DPI-4452 can rapidly provide exceptional images in patients with ccRCC without clinically significant toxicity. High SUVs and tumor-to-background ratios suggest potential for use in both diagnostics and patient selection. These first-in-human findings with radiolabeled DPI-4452 are encouraging for the subsequent evaluation of [^177^Lu]Lu-DPI-4452 treatment.

## DISCLOSURE

Anna Pokorska-Bocci, Solen Pichereau, Jonathan Wessen, Olena Vlasyuk, and Ivana Galetic are employees of Debiopharm International SA. Michael Hofman reports grants (to Peter MacCallum Cancer Centre) from Novartis, ANSTO, Bayer, Isotopia, and MIM and consulting fees from Astellas, AstraZeneca, Janssen, Merck/MSD, and Mundipharma, unrelated to this work. Darren Feldman reports grants/contracts from Decibel Inc. and Telix and personal fees from BioNTech, Renibus Therapeutics, Telix, and Xencor, unrelated to this work. No other potential conflict of interest relevant to this article was reported.

## Notes

KEY POINTS **QUESTION:** Can [^68^Ga]Ga-DPI-4452 be used to visualize ccRCC lesions, and is it safe? **PERTINENT FINDINGS:** [^68^Ga]Ga-DPI-4452 showed rapid and sustained lesion-specific uptake, with optimal visualization 1 h after administration. Pharmacokinetic analysis demonstrated rapid systemic elimination of [^68^Ga]Ga-DPI-4452, and no clinically significant safety findings were observed. **IMPLICATIONS FOR PATIENT CARE:** CAIX is aberrantly expressed in more than 90% of ccRCC cases, and current diagnostic methods using radiolabeled anti-CAIX antibodies take several days to visualize tumors. [^68^Ga]Ga-DPI-4452 is safe, is rapidly cleared from the body, and offers outstanding imaging with same-day administration.
